# Decoherence control by quantum decoherence itself

**DOI:** 10.1038/srep09796

**Published:** 2015-06-11

**Authors:** Katarzyna Roszak, Radim Filip, Tomáš Novotný

**Affiliations:** 1 Department of Theoretical Physics, Wrocław University of Technology, 50-370 Wrocław, Poland; 2 Department of Condensed Matter Physics, Faculty of Mathematics and Physics, Charles University, 121 16 Prague, Czech Republic; 3 Department of Optics, Palacký University, 17. listopadu 1192/12, 771 46 Olomouc, Czech Republic

## Abstract

We propose a general approach of protecting a two-level system against decoherence via quantum engineering of non-classical multiple superpositions of coherent states in a non-Markovian reservoir. The scheme surprisingly only uses the system-environment interaction responsible for the decoherence and projective measurements of the two-level system. We demonstrate the method on the example of an excitonic qubit in self-assembled semiconductor quantum dots coupled to the super-Ohmic reservoir of acoustic phonons.

Decoherence is the most significant obstacle of expanding quantum technology. It appears as a result of an interaction of the quantum system of our interest with an environment^1^,^2^,^3^. The most common source of the decoherence is dephasing reducing a quantum superposition between the eigenstates of energy of the system. If the environment, at least partially, resolves the basis states of the system, their superposition is degraded or, ultimately, it completely vanishes^4^. Frequently, the environment is not directly controllable or measurable, it can be manipulated only by the same interaction causing the decoherence which may represent a serious limit. On the other hand, the system-environment interaction can produce quantum entangled states between the system and the environment^5^. The decoherence becomes a quantum process which can be in principle inverted, as opposed to the classical decoherence^6^,^7^. However, without a direct access to the environment, the reversibility is not feasible. Yet, quantum decoherence can still be used to *pre-engineer*^8^,^9^ the environment to a state which does not cause so destructive decoherence.

As a very good practical example, we can consider semiconductor quantum dots (QDs), zero-dimensional nanostructures, in which charge carriers display a discrete energy spectrum. A vast drawback for many applications of semiconductor QDs is the carrier-phonon interaction which leads to dephasing of electronic superpositions on picosecond time scale^10^,^11^,^12^. To overcome this difficulty, a number of solutions were proposed, including qubits coded on spin states^13^,^14^, hybrid spin-charge schemes^15^,^16^, modification of the optical-pulse shape^17^,^18^ or reservoir properties^19^,^20^, and collective encoding^21^,^22^. Despite some quite promising results have been shown, a substantial reduction of decoherence is accompanied by either amassing *difficulty* in coherent control of the qubit (or many qubits), or by making the ensemble more involved and resulting in fabrication problems. Here, we propose an inhibition of dephasing by reservoir pre-engineering assisted by the same quantum dephasing process via repeated measurements of the qubit state.

## Results

### Quantum dephasing: toy model

The simplest mechanism of *quantum dephasing* for a single energy-degenerate qubit can be described by an interaction with a single environmental quantum oscillator *E* with vanishing frequency distinguishing between computational basis states |0〉 and |1〉 of the qubit. The interaction can be modelled by the interaction Hamiltonian *H*_*I*_ = *κ*|1〉〈1|*P*_*E*_, where *κ* is the interaction constant and 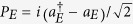 is the momentum operator of the environmental oscillator (we use *ћ* = 1 throughout the paper). The interaction performs a non-demolition monitoring of one of the degenerate states of the qubit, which does not change the equal probabilities of the states |0〉 and |1〉 and only influences their superposition. In this case, the evolution operator 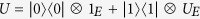 acting on both the qubit and the oscillator *E* generates, if the qubit is in the state |1〉, the unitary transformation of the environmental states *U*_*E*_(*α*) = exp(*iκτP*_*E*_), with 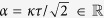, corresponding to coherent displacement along the coordinate variable 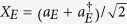. For the environmental oscillator being initially in the ground state 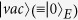, the unitary *U*_*E*_ changes |vac〉_*E*_ to an overlapping coherent state 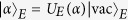. If the testing qubit is initially in the superposition state 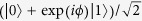, an entangled state 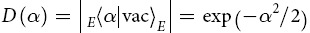 arises between the qubit and the environment. The square-root 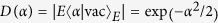 of the overlap between the states of the environment then quantifies both the amount of entanglement and phase damping process transferring the initial qubit state to a mixture 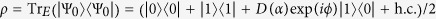.

The entanglement generated by the dephasing can be exploited for the state preparation of the environment. Consider a qubit being prepared initially in the state 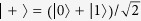. After it has undergone interaction with the environment for the duration *τ*, the projection 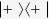 (consisting of the standard *π*/2 -pulse on the qubit system followed by a projective measurement in the basis of ground/excited states) is executed on this qubit^23^. The environment *E* is then projected to the superposition state 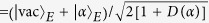. The environment is thus engineered in a nonclassical quantum state being a superposition of non-orthogonal states, known as the *cat state*^24^,^25^. To test, whether the superposition state 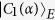 present in the environment can be better for a storage of the qubit, the testing qubit only carrying information in the phase variable *ϕ* is interacting during time interval *t* with the pre-engineered environment *by the same type of interaction* described by *H*_*I*_. The resulting entangled state (
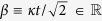
)
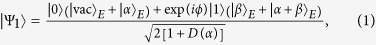
between the qubit and the environment is still subject to the quantum dephasing. However, the overlap of 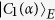 and 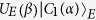 is now substantially different from *D*(*α*). Tracing out the environment, the qubit is then described by the density matrix with the phase damping factor
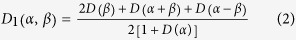
fully characterising the dephasing process after engineering of the environment. The last two terms arise due to interference effects between the state preparation and the subsequent dephasing of the testing qubit. If *α* = *β*, then *D*(*α* *−* *β*)=1 by definition. On the other hand, since *D*(*α*), *D*(*β*) and *D*(*α* + *β*) vanish for large *α* and *β*, the dephasing factor can interestingly converge to *D*_1_ = 1/2 for large equal interaction times *τ* = *t*. This should be contrasted with 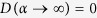 for the initially ground state of the environment.

This is a remarkable result, since by a conditional engineering of the environment using the same quantum dephasing process, we are able to protect the subsequent qubit evolution against the very same dephasing mechanism. The protection arises due to a quantum interference term *D*(*α* *−* *β*) in equation (2) caused by the principal indistinguishability of the state 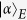 being a component in both the states 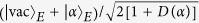 and 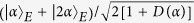 induced by the dephasing interaction for *τ* = *t* in the environment. Is the superposition in the environment really required? Imagine that the engineered superposition collapses into the incoherent mixture 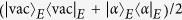 before the testing qubit interacts with the environment. The dephasing factor remains 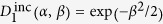, the same as without any environment engineering. Therefore, the quantum superposition of (non-orthogonal coherent) environmental states becomes a *resource* necessary for our method of protecting qubits. Quantum dephasing therefore has the principal feature which allows to be corrected by itself, differently from the classical dephasing.

For the initial ground state of the environment, after *M* identical repetitions of the state preparation with preparation times *τ*, the state superposition 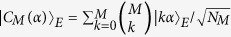 of the environmental coherent states is generated. This special state, a superposition of equidistantly displaced states with the coefficients proportional to the combinatorial numbers from the Pascal triangle, is a direct outcome of the *quantum random walk* with coherent states in the environment and yields for the decoherence factor 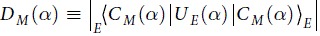 the expression
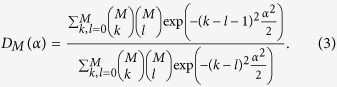


For small 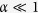 and large 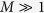, due to overlaps of the states 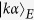 the state 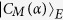 approaches a pure Gaussian state squeezed in the momentum variable *P*_*E*_ with the variance of the momentum 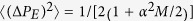 calculated in the Methods section. Consequently, 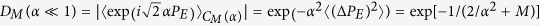, which is increasing with *M*. The measurement-induced squeezing of the reservoir momentum *P*_*E*_ explains why the interaction 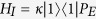 causes less dephasing of the qubit, since the variable *P*_*E*_ is less fluctuating. As shown numerically in the Methods section the above formula approximates equation (3) very well even for large *α*’s and we find the asymptotic behaviour for sufficiently large 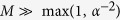

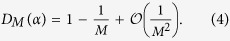


This result implies that the dephasing process can be completely stopped by the repeated state engineering based on the system-environment interaction which is itself responsible for the dephasing. From this perspective, some types of the decoherence processes can be more easily corrected, without any external dynamical operations with the environment, comparing to others, more destructive ones. It opens the broad possibility of further investigations, various extensions and refining of operational understanding what the decoherence actually is about. However, it is unclear whether properties of this simplistic case carry over to more realistic situations involving non-degenerate qubits and environments with a large number of finite frequency modes. As we show in detail below, the answer is positive and we identify a whole class of experimentally-relevant solid-state setups where an analogous mechanism of decoherence suppression can be implemented.

### Infinite reservoir model & its free dynamics

The system under study consists of a self-assembled, single level quantum dot under the influence of a reservoir of longitudinal acoustic phonons described by 
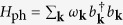
, with *ω*_*k*_ = *vk* being the frequency of the phonon mode with the wave vector **k** (*v* is the speed of longitudinal sound waves). We consider just two electronic states of the dot forming the qubit: |0〉 when the dot is in its ground state (“empty”, i.e. no exciton) and |1〉 indicating the excited QD (“occupied” with an exciton in its ground state) with bare excitation energy 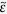, i.e., 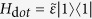. When occupied by the exciton, the dot experiences the interaction with the phonon environment by means of the deformation potential coupling^26^,^27^ 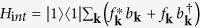 with the super-Ohmic spectral density 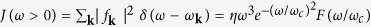 characterised by the low-frequency coefficient 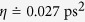, size-dependent high-frequency cut-off 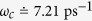, and “form-factor” 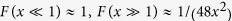 corresponding to the typical material and spatial parameters for a self-assembled InAs/GaAs structure found in Ref. 28 with anisotropic Gaussian exciton wave functions of 5 nm width in the *xy* plane and 1 nm along *z* (for details see the Methods section). The exciton-phonon interaction term in the Hamiltonian is linear in phonon operators and describes a shift of the lattice equilibrium induced by the presence of a charge distribution in the dot associated with the classical energy of the displaced oscillators 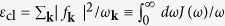. The total Hamiltonian 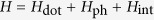 being a variant of exactly-solvable independent boson models is diagonalised^27^ by a canonical transformation represented by the unitary operator 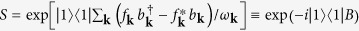 yielding 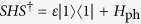, with renormalised (physical) exciton energy 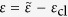 taken equal to 1 eV.

The dynamics of the quantum dot represented by its reduced density matrix 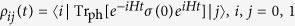 can be solved exactly for factorising initial conditions 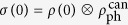 with a canonical state of the phonon reservoir 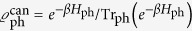 at inverse temperature *β* = 1*/k*_*B*_*T*. Diagonal elements are constant 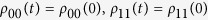, i.e., there is no phonon-induced exciton relaxation, while the time evolution of the off-diagonal elements 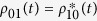 describing the decoherence of superposition states between 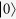 and 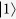 exhibits non-exponential, i.e., non-Markovian decay 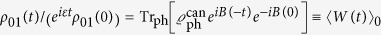 with the Weyl operator^29^,^30^ 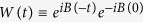. Its equilibrium mean value 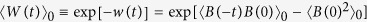
27,31 is governed by the bath correlation function
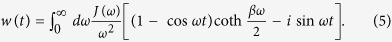


The model thus shows features of *pure dephasing*, i.e., only the coherences, which can be measured by the amplitude of coherent dipole radiation emitted by the dot, decay with time. Moreover, for the super-Ohmic spectral density characteristic of this system, due to the Riemann-Lebesgue lemma the decay saturates at a finite value 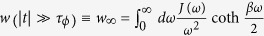 for times much longer than the dephasing time 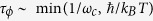, thus the pure dephasing is only *partial* or *incomplete*^29^,^32^,^33^. In the zero-temperature limit 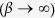 the asymptotic value of the coherence reads 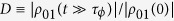 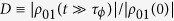

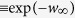
 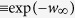

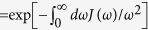
 
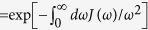

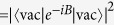
 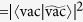, where 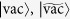 are the phonon vacua when the QD is empty or occupied, respectively. The overlap of the two mutually displaced vacua is non-zero, which means that despite of the continuous spectrum of phonon modes the orthogonality catastrophe is incomplete — this reflects the asymptotic nature of the couplings *f*_***k***_ for small **k**’s (and *ω*) due to identical phonon coupling to electrons and holes for long phonon wavelengths^34^ resulting in the super-Ohmic spectral density of exciton-phonon coupling. Consequently, for small **k**’s the trace left by the exciton in the bath is too weak to be distinguished from the vacuum case and, thus, decoherence is only partial^4^.

### Repeated initialisations

We may study not only the state of the QD considered so far but also the state of the phononic subsystem analogously to the above toy model. The creation of an exciton in the QD perturbs the phonon reservoir state by shifting the coordinates. If the exciton is created in a superposition state, the phonon reservoir will react by following in parallel two different evolutions coherently superposed^35^,^36^. Now, we may ask again, what is the effect of repeated measurements of the dot state on the degree of the partial pure dephasing. Therefore, we analyse the evolution of the composite system of the dot and the phonon reservoir subject to strong projective measurements^23^ performed on the QD subsystem. Each measurement is represented by orthonormal projection operators of the form 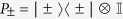 with complementary and orthonormal pure qubit states 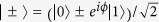 and the unity in the reservoir subsystem 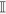. We consider free evolution of the composite system starting from a factorised initial/re-initialised condition 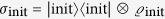 corresponding either to the true initial condition or to an output of previous measurement (see equation (6) below) with the initial state of the QD qubit 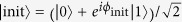 and an arbitrary phonon reservoir density matrix 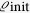. We choose the equal-weight superposition so that neither the dephasing interaction nor the measurement processes, regardless of their outcome, change the occupation factors and therefore, they only influence the coherences.

Under these assumptions the state of the composite system right after the measurement at time *τ* with the outcome ± is given by
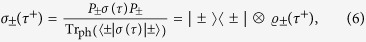
with the measurement-outcome-dependent phonon reservoir density matrices
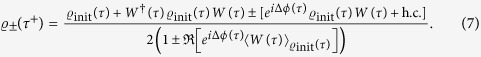
Here, 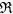 denotes the real part, *h.c.* the hermitian conjugate, 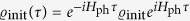, 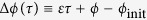, and 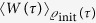 denotes the average of the Weyl operator with respect to this time-evolved phonon density matrix. The respective measurement outcomes are obtained with probabilities 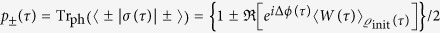.

Note furthermore, that regardless of the measurement outcome, the degree of coherence just after the measurement is fully restored to unity 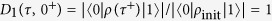, i.e., the net outcome of the measurement on the state of the qubit is, apart from a possible (controlled) phase shift, just the re-initialisation of the qubit state (compare with equation (2) for 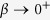). However, the state of the phonon reservoir does change and this has important consequences for further evolution of the qubit. The scheme outlined above can be iterated to yield results for an arbitrary series of measurements, but it acquires great complexity rapidly with the growing number of measurements. It is therefore convenient to study just the single-measurement scenario, especially since an observable decrease of dephasing can be detected already there.

## Discussion

To examine the consequences of repeated initialisation, we study time evolution of the qubit at time *t* after a single measurement performed time *τ* after its initialisation. In particular we monitor the degree of coherence 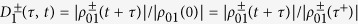 as functions of the delay time *t*, measurement time *τ* and the measurement outcome (±). To this end, we evolve the density matrices from equation (6) for the time span *t* and then evaluate the coherences 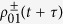. Calculation follows the line analogous to the free evolution discussed above with the initial thermal density matrix 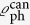 replaced with those of equation (7) leading to 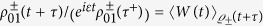. Using the fact that with 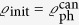 we also get 
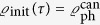
, the result reads (for details, see the Methods section)
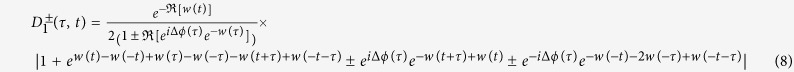
This result is proportional to 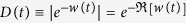 which means that the asymptotic value for large times *t* can only be nonzero if 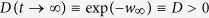, i.e., for partial dephasing, as is the case of the super-Ohmic bath. We then get for large times 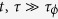 (we use 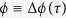)
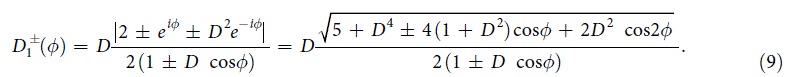


**Figure 1:**
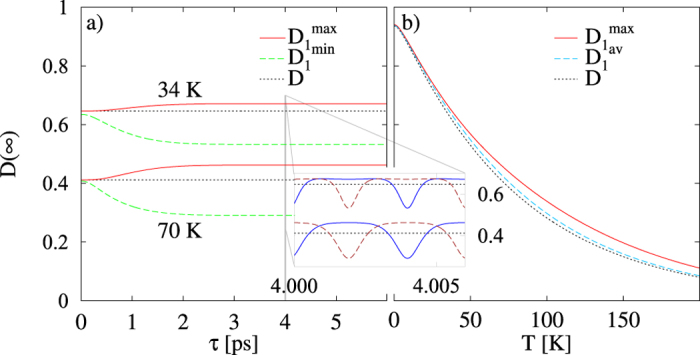
**a**) Asymptotic degree of coherence as a function of the delay time for two temperatures. The envelopes of the maximal (solid red line) and minimal (dashed green line) values of 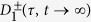 (8) are shown together with the detailed time evolution for the measurement outcome 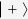 (solid blue line) and 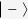 (dashed brown line) on a much shorter time scale in the inset. **b**) Maximal value (full red line) as well as the averaged one (dashed cyan line; see the main text for details) of the asymptotic degree of coherence for a range of temperatures. In both panels, the dotted orange lines denote the degree of coherence in the measurement-free case.

Obviously, these values oscillate as functions of the delay time *τ* between the preparation of the qubit and its measurement with the frequency determined by the shifted exciton energy *ε* (corresponding period is on the order of few femtoseconds) as depicted in the inset of Fig. 1. We also plot there the envelopes of curves (9) on the longer timescale of picoseconds showing the saturation of the initial sub-picosecond transient behaviour. The overall magnitude of the asymptotic degree of coherence decreases with increasing temperature as presented in Fig. 1b). Let us now analyse the formulas (9) in more detail. First, *D*^−^ is easily obtained from *D*^+^ by the phase shift 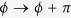 so that it suffices to study the latter one. It always attains a minimum *D*(1 + *D*)/2 at 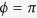 and has a local extremum 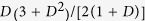 at 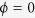. For small enough 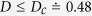 (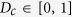 is determined by 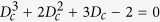) this extremum is the global maximum, while for larger 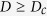 it is just a local minimum and the maximum 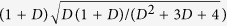 is realised at 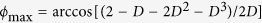. The difference of the maximal value from the free case value *D* is maximised for 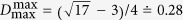 (corresponding to 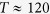 K) by the excess value of 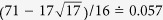, some 20% above the free case. As mentioned in the Methods section we may be also interested in the weighted average 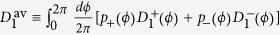 which is bigger than *D* since the integrand is never below *D* (equality happens only at *ϕ* = 0,*π*). Numerical analysis reveals that the maximum difference from the free case is obtained at 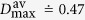 (corresponding to *T* **≈** 60 K) with the magnitude roughly 0.019, about 4% of the free case value. These conclusions are consistent with the plots in Fig. 1b).

Several experiments with self-assembled QDs considered here have been recently realised^37^,^38^,^39^. We have analysed thus far properties of an idealised model and it is necessary to scrutinise whether our conclusions can be carried over to the experimentally realistic situations. There are several points which might in principle endanger our conclusions. First, we have only considered the Hamiltonian describing the free evolution, which is purely harmonic in the acoustic phonon modes and the excitonic interaction with them is solely of pure dephasing type. In reality there are also optical phonons which cause the relaxation of the exciton occupation and, moreover, there is radiative relaxation channel too — these effects, however, become effective only at much longer timescales on the order of tens or hundreds picoseconds^10^ while our asymptotic times are just a few picoseconds. Since the dephasing-suppression mechanism hinges on the creation of “cat states” of the acoustic reservoir modes, their potential dephasing beyond the excitonic interaction by anharmonic terms or by coupling to other (e.g., optical) modes would be detrimental to the predicted effect. While such effects do exist and may be relevant in certain contexts (see, e.g., Ref. 40), the estimated lifetime of the acoustic phonons^41^ is on the order of 1 nanosecond, which makes these issues irrelevant for our discussion. Finally, we have assumed an instantaneous projective measurement of the qubit state. This is clearly not realistic as existing projective measurements are achieved by optical pulses whose duration is at least ten(s) femtoseconds during which the freely evolving qubit phase *ετ* acquires several multiples of 2*π*’s (see the inset of Fig. 1). Thus, one might expect that the effect would be smeared by the phase averaging. However, finite duration of pulses is not necessarily fatal to our predictions. What matters is the short duration of the pulse with respect to the characteristic time scale of the *phonons* being on the order of 1 ps (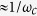) and the ability to very precisely control the relative phase between the initialisation and measurement pulses. This is currently possible by splitting the initial pulse and using the optical delay line with exquisite sub-cycle tuning of the relative phase as realised in pump-probe and multidimensional optical spectroscopies^10^,^42^. Thus, the approximation of delta-like pulses is done and justified for the study of phonon dynamics^12^. Even if the experiment is not completely controlled (the relative phase *ετ* is fluctuating between subsequent runs of the measurement) and/or the measurement outcomes of the qubit state are ignored (e.g., to avoid discarding data), the averaged result described by the quantity 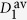 introduced above and plotted in Fig. 1b) still shows enhancement over the free case, although its magnitude is 3-times less (on the absolute scale) than in the fully controlled case. Altogether, we believe that the predicted effect should be experimentally observable.

To summarise, we have proposed measurement-induced quantum pre-engineering of a non-Markovian environment consisting of a super-Ohmic reservoir of longitudinal acoustic phonons which can be directly exploited to control quantum-dot-based qubit decoherence using only the single type of coupling between the qubit and the environment. A feasible proof-of-principle experimental test of the proposed method with self-assembled semiconductor quantum dots would be a practical test of the quantum nature of dephasing for a solid state system. The method can also be translated to the cavity QED, atomic, or trapped ion experiments.

## Methods

### Environment engineering by many repetitions; derivation of equation (3)

After the *M*-times identical state preparation with the preparation times *τ* and subsequent evolution of the qubit during the same time *τ*, the phase damping factor is defined as an absolute value of the scalar product between states
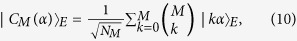
and the displaced version
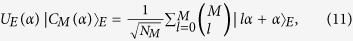
with the same normalisation factor 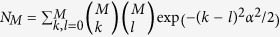. The coherent states 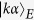 with vanishing mean of momentum *P*_*E*_ can be expressed in the coordinate representation of 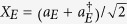 operator in the form of
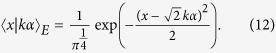


In the limit of small 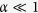 the constituents of the sums (10) and (11) highly overlap and form smooth resulting wave functions. Moreover, at large 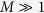 we can approximate the binomial coefficients by expansion based on the Stirling formula 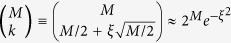 yielding for the normalisation factor
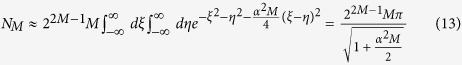
and, similarly, for the whole wave function
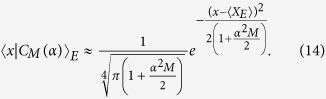


**Figure 2:**
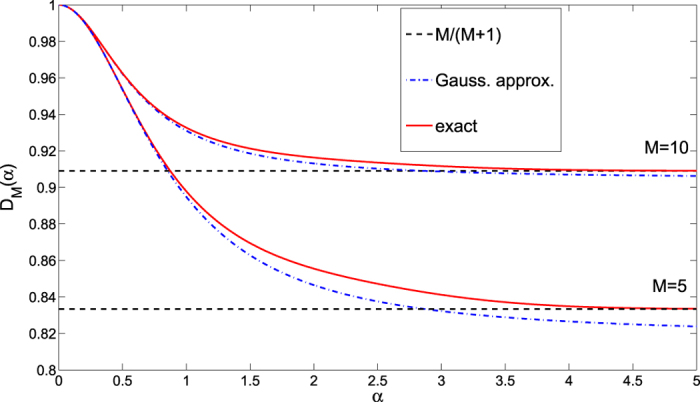
Asymptotic decoherence factor 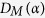 for two values of *M* = 5 (lower set of curves) and *M* = 10 (upper curves) as functions of the integrated interaction strength *α*. Exact expression (3) (full red lines) is compared to the Gaussian approximation (16) (blue dash-dotted lines) and the asymptotic value (17) (black dashed lines).

It is a pure Gaussian state in the environment with the mean 
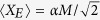
 and variance 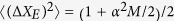. Consequently, the variance in the momentum reads 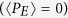

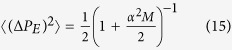
as stated in the main text. This description based on the pure Gaussian state in the environment giving
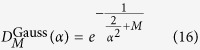
very satisfactorily approximates the exact numerical evaluation of equation (3) for small enough 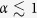 as we show in Fig. 2.

On the other hand, in the limit of large *α*, the coherent states |*ka*〉_*E*_ become almost orthogonal for different *k*’s and we can treat them approximately as the basis states. We can therefore approximate the scalar product 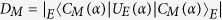 by
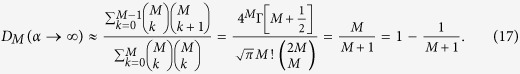


It has the form of equation (4) and approximates the dephasing factor *D*_*M*_ obtained numerically for large 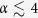 very well as also seen in Fig. 2.

### Parameters used in the model QD Hamiltonian

Carrier-phonon interaction constants *f*_***k***_ in 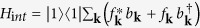 are given by (in this part we reinsert 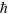 into the expressions)
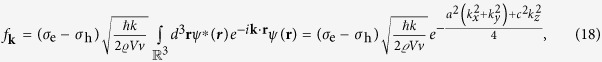
where 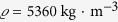 is the crystal density, *V* is the volume of the phonon system, 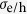 (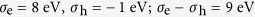) are deformation potential constants for electrons and holes, *v* = 5100 m s^−1^ speed of longitudinal sound waves^28^, and 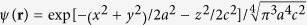 are the exciton wave functions modelled by anisotropic Gaussians with *a* = 5 nm width in the *xy*-plane and *c* = 1 nm along the *z*-axis. Therefore, we get for the spectral density (recall that 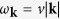)
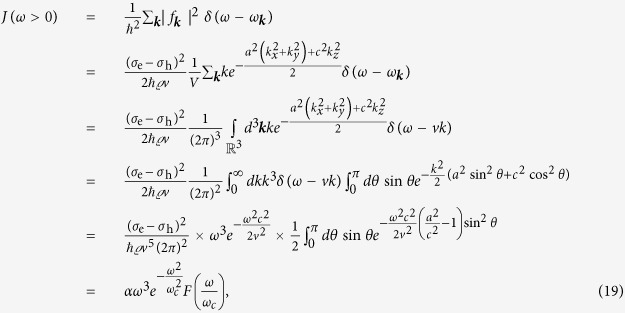
with the coefficient 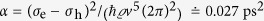, cut-off frequency 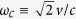 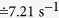
and the function *F*(*x*) given by the last integral expression whose asymptotic behaviour for small and large *x* is stated (for *a*/*c* = 5) in the main text.

### Derivation of equations (7) and (8)

As mentioned in the main text the time evolution 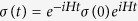 of a factorising initial state of the qubit plus the phonon environment in the form 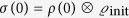 can be solved formally exactly by employing the Weyl operator 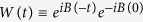 following the chain of arguments (recall that 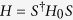 with 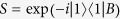 and 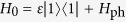)
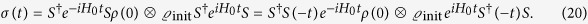
From the definition of *S* we get 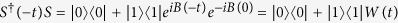 and, using the initial pure state of the qubit 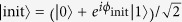, we can write for *σ*(*t*) in the block matrix form in the qubit basis 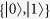

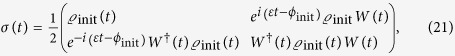
with 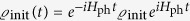. The projective measurement onto states 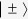 at time *τ* then yields equation (6) with the (normalised) phonon bath density matrices 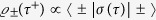 stemming from equation (21) (with the help of relation 
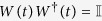
) given in equation (7).

We can use equation (21) also for the subsequent time evolution of the density matrix for time *t* after the measurement via replacing 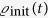 by 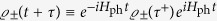 since the total state of the system plus phonon reservoir just after the measurement (6) is of the factorised form assumed in its derivation. Consequently, we obtain for the off-diagonal element of the qubit density matrix 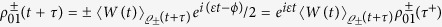 and the degree of decoherence is determined by the quantity
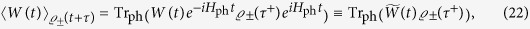
with 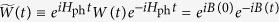. Using the fact that the very initial state of the phonon reservoir was canonical and, therefore, also 
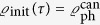
 we can write (recall that 
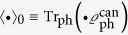
)
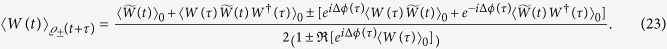


The required mean values are calculated with the help of cumulants (due to the Gaussian nature of the canonical density matrix the second cumulants give *exact* results — see, e.g., Ref. [27])
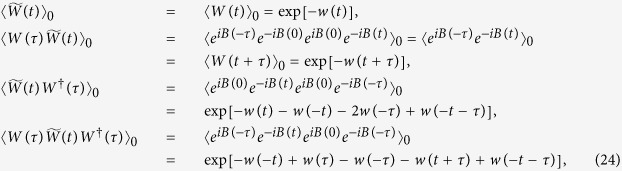
which eventually yields equation (8).

## Additional Information

**How to cite this article**: Roszak, K. *et al.* Decoherence control by quantum decoherence itself. *Sci. Rep.* **5**, 09796; doi: 10.1038/srep09796 (2015).
